# Primary bivalirudin anticoagulation for patients with an implantable ventricular assist device

**DOI:** 10.1186/cc13290

**Published:** 2014-03-17

**Authors:** M Pieri, B Arnaez, Al Di Prima, M Celinska-Spodar, S Ajello, O Saleh, F Isella, A Montisci, F Pappalardo

**Affiliations:** 1 San Raffaele Scientific Institute, Milan, Italy

## Introduction

Bivalirudin is a direct thrombin inhibitor that is increasingly used in patients undergoing mechanical circulatory support as it presents many advantages compared with heparin. The aim of this study was to describe our experience with bivalirudin as primary anticoagulant in patients undergoing ventricular assist device (VAD) implantation.

## Methods

An observational study on the 12 consecutive patients undergoing VAD implantation from October 2011 at out institution. Five patients were implanted the Heart Mate II LVAD (Thoratec Corp., Pleasanton, CA, USA), six patients the Heartware HVAD (HeartWare Inc., Miramar, FL, USA), and one patient a Cardiowest Total Artificial Heart (Syncardia Systems Inc., Tucson, AZ, USA). Patients received a continuous infusion of bivalirudin, with a starting dose of 0.025 μ/ kg/hour. The target activated partial thromboplastin time (aPTT) was between 45 and 60 seconds.

## Results

Patients never received heparin during hospitalization nor had a prior diagnosis of HIT. Preoperative platelets count was 134,000 ± 64,000 platelets/mm^3^. The bivalirudin dose was 0.040 ± 0.026 mg/kg/hour, and the duration of therapy was 5 (5 to 12) days. The lowest platelet count during treatment was 73,000 ± 23,000 platelets/mm^3^. No thromboembolic complications occurred. Two episodes of minor bleeding from chest tubes which subsided after reduction or temporary suspension of bivalirudin infusion were observed. Six patients required blood red cell transfusions, and one patient had one fresh frozen plasma transfusion. No platelet transfusions were performed during treatment. The ICU stay was 8 (7 to 17) days, and the hospital stay 25 (21 to 33) days.

## Conclusion

Bivalirudin is a valuable option for anticoagulation in patients with VAD, and can be easily monitored with aPTT. The use of a bivalirudin-based anticoagulation strategy in the early postoperative period may overcome many limitations of heparin, and above all the risk of HIT which is higher in patients undergoing cardiac surgery. Bivalirudin should no longer be regarded as a second-line therapy for anticoagulation in patients with VAD.

